# *Bifidobacterium lactis* TY-S01 Prevents Loperamide-Induced Constipation by Modulating Gut Microbiota and Its Metabolites in Mice

**DOI:** 10.3389/fnut.2022.890314

**Published:** 2022-06-29

**Authors:** Tian Tang, Jing Wang, Yuanyuan Jiang, Xu Zhu, Zhen Zhang, Yuying Wang, Xi Shu, Yadan Deng, Feng Zhang

**Affiliations:** Chongqing Tianyou Dairy Co., Ltd., Chongqing, China

**Keywords:** probiotics, constipation, gut microbiota, SCFAs, 5-HT, inflammation

## Abstract

Probiotics have received widespread attention as a healthy ingredient. The preventive effect of *Bifidobacterium lactis* TY-S01 on loperamide-induced constipation in mice was investigated in this study. TY-S01 accelerated the peristalsis of intestine, maintained the humidity of faeces, and prevented the destruction of gut barrier. TY-S01 also maintained the 5-HT, MTL and SP at normal levels in constipated mice. Simultaneously, TY-S01 up-regulated the mRNA expressions of 5-HT_4_R, SERT, and MUC-2, while down-regulated the mRNA expressions of pro-inflammatory genes remarkably. The levels of short-chain fatty acids in the feces of constipated mice were also increased because of the intervention with TY-S01. Moreover, TY-S01 prevented gut microbiological dysbiosis in constipated mice. Spearman’s correlation analysis revealed that there was an obvious association between metabolic biomarkers and gut microbiota. In summary, TY-S01 regulated gut microbiota and the production of intestinal metabolites to prevent loperamide-induced constipation.

## Introduction

Constipation is a prevalent gastrointestinal disease characterized by reduced stool volume, dry stools, difficulty defecation, and infrequent bowel movements (1). Drugs are frequently utilized to treat constipation currently, however, the therapy gives rise to many complications such as severe diarrhea and drug dependence (2). Hence, efficacious treatments without side effects are being expected.

The latest research has revealed that dysbiosis of gut microbiota increases constipation. Decreased abundance of beneficial bacteria while the excessive abundance of opportunistic pathogens in the gut of constipated individuals has been reported (3). The inflammatory response, impaired intestinal mucosal barrier, reduced intestinal metabolites, intestinal dysfunction, abnormal neurotransmitter, and neurochemical signaling caused by the dysbiosis of gut microbiota can decelerate intestinal motility, thereby contributing to constipation (4–6). Intestinal metabolites short-chain fatty acids (SCFAs) are beneficial, which promote intestinal motility by reducing intestinal pH and inhibiting inflammation (7). The 5-hydroxytryptamine (5-HT) is mainly synthesized in the gut and is a pivotal neurotransmitter of the brain–gut axis (8). 5-HT receptor 4 (5-HT_4_R) is a specific receptor for 5-HT, which contributes to ameliorating constipation (9). Serotonin transporter (SERT) is a transmembrane transporter responsible for transporting 5-HT (2). Therefore, the down-regulated expression of SERT and 5-HT_4_R predicts intestinal dysfunction.

Probiotics, including *Bifidobacterium* and *Lactobacillus*, have been confirmed to have multiple health benefits. Recent studies suggested that probiotics could influence the development of constipation by affecting the intestinal transit time, the stool frequency and consistency, and the gut microbiota (10). Agrawal et al. put forward that *Bifidobacterium lactis* DN173010 shortened the time of intestinal transit by an average of 12.2 h (11). The research by Yang et al. determined that dietary synbiotics (*Bifidobacterium animalis* subsp. *lactis*, *Lactobacillus rhamnosus*, *Lactobacillus acidophilus*, and *Lactobacillus plantarum* participated in the composition) alleviated constipation *via* increasing the population of beneficial bacteria, stimulating the emancipation of gastrointestinal hormones and repressing the inflammatory response in mice (12). Not exclusively, another report verified that *Bifidobacterium* heightened the population of *Lactobacillus* but lowered the population of *Odoribacter* and *Clostridium* in the gut to attenuate constipation in mice, and illustrated interspecies differences (13). Wang et al. proposed that *B. animalis* subsp. *lactis* MN-Gup relieved constipation by enhancing the levels of intestinal SCFAs (14). Furthermore, the emancipation of 5-HT was promoted and the expressions of SERT and 5-HT_4_R were up-regulated after the supplementation of *Lactobacillus paracasei* X11 and prebiotics in constipated mice (2). Probiotics are “strain-specific” in their efficacy. Therefore, the selection and breeding of probiotics with excellent performance in preventing constipation will contribute to enriching probiotic resources and provide more personalized choices.

In this study, we provided a probiotic strain, *B. lactis* TY-S01, and surveyed its preventive effect on loperamide-induced constipation of mice. Furthermore, the potential mechanism of TY-S01 to prevent constipation was explored.

## Materials and Methods

### Materials

*Bifidobacterium lactis* TY-S01 was separated from the intestine of long-lived elderly in Bama, Guangxi, China, and was preserved in the China General Microbiological Culture Collection Center (Beijing, China) with the accession No. 21255. The colony number was adjusted to 1.0 × 10^9^ CFU/kg.BW before gavage.

### Animal Experiments

The animal experimental scheme was authorized by the Experimental Animal Welfare Ethics Review Committee of Chongqing Institute of Traditional Chinese Medicine (5001087226041, Chongqing, China). Seven-week-old BALB/c male mice were housed in a standardized laboratory and fed under standard conditions. The experiment was started after 1 week of adaptive feeding. Mice were divided into three groups randomly, namely control (NC), constipation model (CM), and TY-S01 intervention (TY-S01) group containing 10 mice each. The experiment was divided into two periods, the TY-S01 intervention period and constipation modeling period, respectively, and the model design and the dosage of loperamide referred to the method of Wang et al. (15). In the TY-S01 intervention period (1–14 days): the NC group and CM group were given normal saline while the TY-S01 group was given 10^9^ CFU/kg.BW bacterial solution. In the constipation modeling period (15–17 days): all of the groups were gavaged 10 mg/kg.BW loperamide except for the NC group, the mice of the NC group were given normal saline. The mice were administered again as similar to the TY-S01 intervention period after an interval of 1 h. The gavage volume was 10 mL/kg.BW throughout the whole experiment. On the 17th day, all mice were fasted for 16 h after the gavage and then given 10 ml/kg.BW of activated carbon solution (18th day). Five mice in each group were used to measure the time of the first black stool (FBS), and the remaining five mice were used to measure the gastrointestinal transit rate (GTR). All mice had free access to water and food during the experiment, and the weight of the mice was recorded.

### Sample Collection

The feces of mice in each group on day 17 were collected for the determination of fecal water content (FWC) and fecal SCFAs level. After the experiment, blood was collected, and the serum was obtained after refrigerated centrifugation at 3,000 *g* for 15 min for biochemical analysis and metabolomics analysis; small intestine and colon tissues were collected for histological observation and real-time PCR; the cecal contents of each mouse were collected for microbiome analysis.

### Measurement of Time of First Black Stool

Mice were placed in a clean cage individually and the time that each mouse excreted its FBS was recorded.

### Measurement of Fecal Water Content

The wet weight of the feces was measured after the defecation of the mice instantly, then the feces were thoroughly dried in an oven and weighed again to obtain the dry weight. FWC was calculated in the light of following formula: FWC(%)=[(*wetweight*−*dryweight*)/*wetweight*]×100%.

### Measurement of the Gastrointestinal Transit Rate

The mice were sacrificed 20 min after gavage with activated carbon and the whole intestines were collected to survey the transit distance of activated charcoal. GTR was calculated in the light of following formula: GTR(%)=(*charcoalmarker*/*intestinallength*)×100%.

### Histological Analysis of the Small Intestine

About 0.5 cm of intestinal segments were cut, washed with saline, and fixed in 4% paraformaldehyde, embedded and paraffin sectioned within 72 h, then the fixed intestinal segments were routinely stained with hematoxylin and eosin (H&E) (2). An inverted fluorescence microscope (Leica DMi8, Weztlar, Germany) was used to observe the tissue state.

### Measurement of the Biochemical Indicators

The levels of 5-HT, motilin (MTL), and substance P (SP) were surveyed in the serum of mice using an enzyme-linked immunosorbent assay kit (Elisa Biotech Co., Ltd., Shanghai, China).

### Measurement of mRNA Expression by RT-PCR

Total RNA from colon tissues was extracted with TRIzol (Invitrogen, CA, United States). cDNA was synthesized by reverse transcription of 1 μg RNA using the RevertAid First Strand cDNA Synthesis Kit (Invitrogen, CA, United States). The level of mRNA expressions was measured by a real-time PCR machine (Bio-Rad, CA, United States). Primer sequences are shown in Table 1 (GAPDH as reference gene). The relative expression of mRNA was computed using the 2^–ΔΔ*CT*^ method.

**TABLE 1:** The sequences of the target gene.

Gene name	5′-3′
5-HT_4_R-F	GATGCTAATGTGAGTTCCAACGA
5-HT_4_R-R	CAGCAGGTTGCCCAAGATG
SERT-F	TATCCAATGGGTACTCCGCAG
SERT-R	CCGTTCCCCTTGGTGAATCT
MUC-2-F	ATGCCCACCTCCTCAAAGAC
MUC-2-R	GTAGTTTCCGTTGGAACAGTGAA
TNF-α-F	CCTGTAGCCCACGTCGTAG
TNF-α-R	GGGAGTAGACAAGGTACAACCC
IL-6-F	TAGTCCTTCCTACCCCAATTTCC
IL-6-R	TTGGTCCTTAGCCACTCCTTC
IL-1β-F	GAAATGCCACCTTTTGACAGTG
IL-1β-R	TGGATGCTCTCATCAGGACAG
GAPDH-F	TGACCTCAACTACATGGTCTACA
GAPDH-R	CTTCCCATTCTCGGCCTTG

### Measurement of the Short-Chain Fatty Acids by GC

About 50 mg of feces were added to saturated sodium chloride solution, followed by acidification with 10% sulfuric acid and extraction with 1,000 μl of ether. The supernatant was added with 0.25 g of anhydrous sodium sulfate, and the supernatant was collected again (16). Chromatography was performed using a GC-7890A (Agilent Technologies Inc., CA, United States) with a flame ionization detector. GC conditions: the initial temperature was 100°C, maintained for 5 min, increased to 250°C at a rate of 10°C min^–1^, and held for 12 min. The injection volume was 1 μl.

### Analysis of the Microbial Diversity

Eight samples of cecal contents were randomly selected from each group for gut microbiota analysis. The ABI GeneAmp^®^ 9700 PCR thermocycler (ABI, CA, United States) was used to amplify the hypervariable region V3–V4 of the bacterial 16S rRNA gene with primer pairs 338F (5′-ACTCCTACGGGAGGCAGCAG-3′) and 806R (5′-GGACTACHVGGGTWTCTAAT-3′). Sequencing was performed using an Illumina NovaSeq PE250 platform (Illumina, San Diego, CA, United States) according to the standard protocols by Majorbio Bio-Pharm Technology Co., Ltd. (Shanghai, China). The data were analyzed on the online platform of Majorbio Cloud Platform.^1^ The representative sequences for the ASVs were annotated using the Bayes annotation method and through the Silva 138/16S-bacteria database. In order to complete the subsequent analysis, sequences were rarefied to the lowest number of sequences per sample (*n* = 25,359 sequences).

### Statistical Analysis

The difference between groups was compared by one-way ANOVA and Tukey’s test using SPSS version 20.0 software. Figures were made using GraphPad Prism 7.0 software. A *p* < 0.05 was considered statistically significant.

## Results

### TY-S01 Prevented Loperamide-Induced Constipation in Mice

**FIGURE 1:**
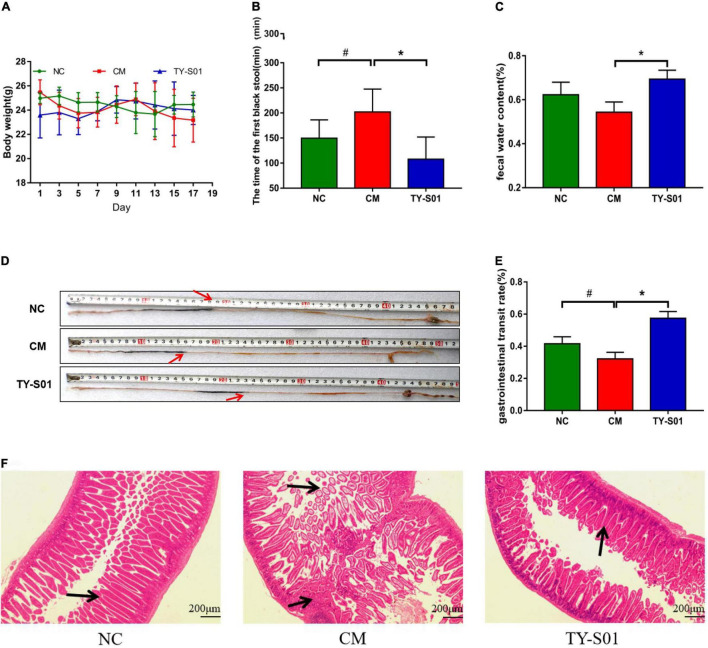
TY-S01 prevented loperamide-induced constipation in mice. **(A)** Body weight; **(B)** the time of the first black stool; **(C)** fecal water content; **(D)** intestinal advancement distance; **(E)** gastrointestinal transit rate; and **(F)** small intestinal sections stained with hematoxylin and eosin. NC is the normal control group; CM is the constipation model group; TY-S01 is the treatment group with TY-S01. ^#^ represents the significant difference between the NC and CM groups (*p* < 0.05); * represents the significant difference between the CM and TY-S01 groups (*p* < 0.05). Data are presented as mean ± SD.

The mice was treated with TY-S01 for 14 days followed by giving with loperamide for 3 days to assess the effect of TY-S01 on constipation. Body weight had no obvious difference in mice during the experiment (*p* < 0.05) (Figure 1A), illustrating that the mice were in good physiological status. The consequence demonstrated that TY-S01 intervention shortened the time of FBS remarkably and maintained the FWC in constipated mice (*p* < 0.05, Figures 1B,C). Besides, the GTR in the CM group was dramatically lower than in the NC group, while increased in the TY-S01 group (*p* < 0.05, Figures 1D,E). Hence, TY-S01 treatment prevented constipation in mice effectively.

The effect of TY-S01 on intestinal morphology was determined in constipated mice by histological staining. H&E staining revealed that loperamide caused the fractured and uncomplete villi in the small intestine, while TY-S01 maintained the integrity of the ileal wall and villi. Furthermore, TY-S01 administration suppressed the lamina propria cell infiltration in the intestine (*p* < 0.05, Figure 1F). The results illustrated that TY-S01 maintained intestinal epithelial integrity in constipated mice.

### Effect of TY-S01 on the Levels of 5-Hydroxytryptamine and Gastrointestinal Hormone in Serum of Mice

**FIGURE 2:**
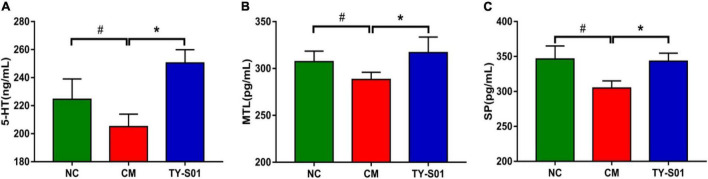
Effect of TY-S01 on the levels of 5-HT and gastrointestinal hormone in serum of mice. The serum level of 5-HT **(A)**, MTL **(B)**, and SP **(C)**. 5-HT is 5-hydroxytryptamine; MTL is motilin; SP is substance P. NC is the normal control group; CM is the constipation model group; TY-S01 is the treatment group with TY-S01. ^#^ represents the significant difference between the NC and CM groups (*p* < 0.05); * represents the significant difference between the CM and TY-S01 groups (*p* < 0.05). Data are presented as mean ± SD.

The effect of TY-S01 on the serum level of 5-HT, MTL, and SP is displayed in Figure 2. The 5-HT, MTL, and SP levels in the CM group were notably reduced compared to the NC group (*p* < 0.05). However, 5-HT, MTL, and SP remained at normal levels after TY-S01 treatment (*p* < 0.05). The results suggested that the mechanism by which TY-S01 prevented constipation was associated with the release of 5-HT and gastrointestinal hormones probably.

### Effect of TY-S01 on Intestinal Function and Intestinal Inflammation of Mice

**FIGURE 3:**
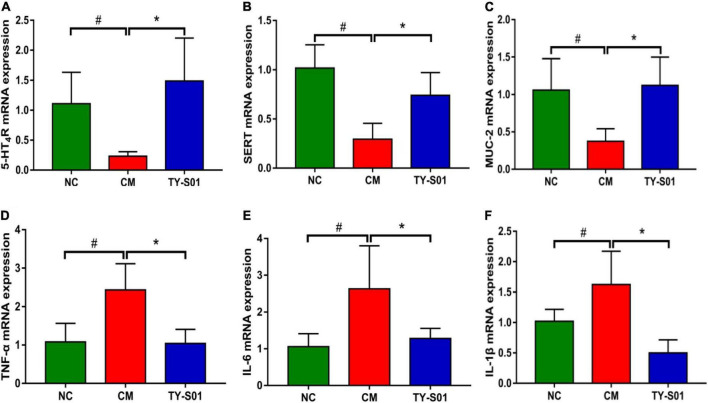
Effect of TY-S01 on intestinal function and intestinal inflammation of mice. The mRNA expression levels of 5-HT_4_R **(A)**, SERT **(B)**, MUC-2 **(C)**, TNF-α **(D)**, IL-6 **(E)**, and IL-1β **(F)**. 5-HT_4_R is 5-HT receptor 4; SERT is serotonin transporter; MUC-2 is mucin 2; TNF-α is tumor necrosis factor-α; IL-6 is interleukin-6; IL-1β is interleukin-1β. NC is the normal control group; CM is the constipation model group; TY-S01 is the treatment group with TY-S01. ^#^ represents the significant difference between the NC and CM groups (*p* < 0.05); * represents the significant difference between the CM and TY-S01 groups (*p* < 0.05). Data are presented as mean ± SD.

The effect of TY-S01 on function, mucosal barrier, and inflammation in the colon of mice was assessed by RT-PCR. In the CM group, the mRNA expression of 5-HT_4_GPCR and SERT was lessened obviously (*p* < 0.05, Figures 3A,B). TY-S01 treatment maintained the mRNA expressions of 5-HT_4_G and SERT at normal levels (*p* < 0.05, Figures 3A,B), which attenuated intestinal dysfunction in constipated mice. The MUC-2 was the element of intestinal tight junction, and the MUC-2 mRNA expression was down-regulated in the CM group but was up-regulated significantly in the TY-S01 group (*p* < 0.05, Figure 3C). Moreover, the mRNA expression levels of TNF-α, IL-6, and IL-1β were decreased in mice of the CM group compared with the NC group, TY-S01 suppressed the expression of TNF-α, IL-6, and IL-1β meaningfully (*p* < 0.05, Figures 3D–F). The consequences proved that TY-S01 attenuated intestinal inflammation in constipated mice.

### Effect of TY-S01 on the Structure of Gut Microbiota in Mice

**FIGURE 4:**
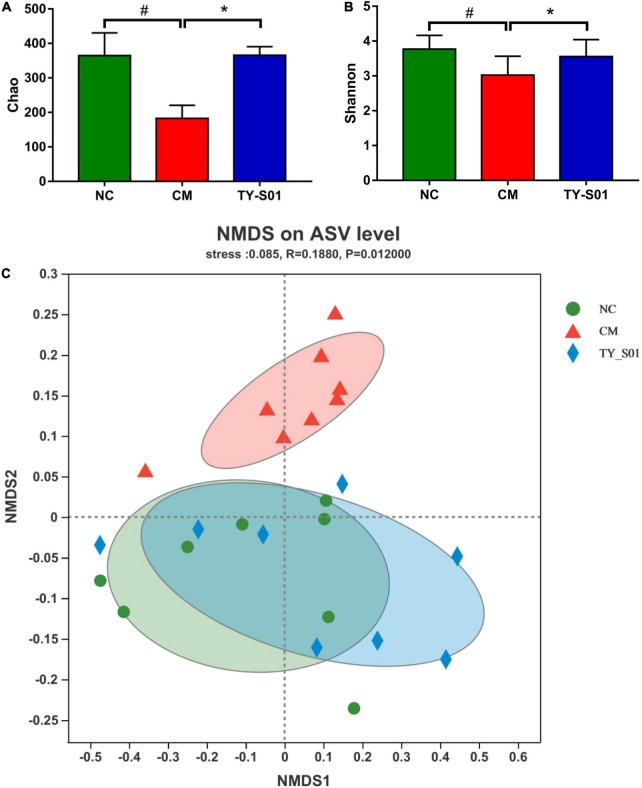
Effect of TY-S01 on the structure of gut microbiota in mice. **(A)** Chao index; **(B)** Shannon index; and **(C)** NMDS at the ASV level. NMDS is non-metric multidimensional scaling; ASV is amplicon sequence variants. NC is the normal control group; CM is the constipation model group; TY-S01 is the treatment group with TY-S01. ^#^ represents the significant difference between the NC and CM groups (*p* < 0.05); * represents the significant difference between the CM and TY-S01 groups (*p* < 0.05). Data are presented as mean ± SD.

The effect of TY-S01 on gut microbiota in constipated mice was explored by 16S rDNA sequencing technology. Chao index and Shannon index are α-diversity indices, which are utilized to assess the richness and diversity of a community, respectively. Chao index and Shannon index were reduced in the CM group, but higher in the NC group and TY-S01 group (*p* < 0.05, Figures 4A,B). The β-diversity between microbiome samples was determined *via* the non-metric dimensional scaling (NMDS), which illustrated that the cluster of the TY-S01 group was similar to the NC group but relatively separated from the CM group (Figure 4C). Therefore, even after loperamide treatment, TY-S01 maintained the gut microbiota of mice in a balanced state.

### Effect of TY-S01 on the Composition of Gut Microbiota in Mice

**FIGURE 5:**
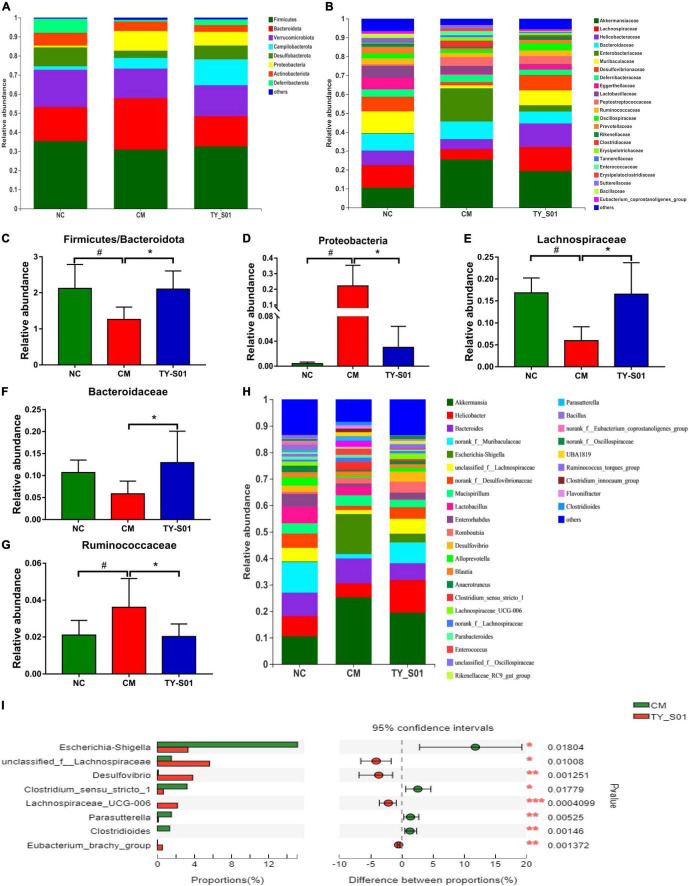
Effect of TY-S01 on the composition of gut microbiota in mice. **(A)** Taxonomic distribution of bacterial communities at the phylum level; **(B)** taxonomic distribution of bacterial communities at the family level; **(C)** the ratio of *Firmicutes* to *Bacteroidetes*; **(D)** the relative abundance of *Proteobacteria*; **(E)** the relative abundance of *Lachnospiraceae*; **(F)** the relative abundance of *Bacteroidaceae*; **(G)** the relative abundance of *Ruminococcaceae*; **(H)** taxonomic distribution of bacterial communities at the genus level; and **(I)** species difference analysis at the genus level. NC is the normal control group; CM is the constipation model group; TY-S01 is the treatment group with TY-S01. ^#^ represents the significant difference between the NC and CM groups (*p* < 0.05); * represents the significant difference between the CM and TY-S01 groups (*p* < 0.05). Data are presented as mean ± SD. *, **, and *** represent 0.01 < *p* ≤ 0.05, 0.001 < *p* ≤ 0.01, and *p* ≤ 0.001, respectively.

The effect of TY-S01 on the abundance of special species in the gut of mice was explored at the phylum, family, and genus levels, respectively. At the phylum level, the ratio of *Firmicutes* to *Bacteroidetes* was increased because of TY-S01 in constipated mice (*p* < 0.05, Figures 5A,C). Meanwhile, the abundance of *Proteobacteria* in the CM group was significantly higher than that in the TY-S01 group and NC group (*p* < 0.05, Figures 5A,D). At the family level, loperamide decreased the populations of *Lachnospiraceae*, *Bacteroidaceae*, and increased the population of *Ruminococcaceae* (*p* < 0.05, Figures 5B,E–G). However, TY-S01 maintained the normal abundance of these species (*p* < 0.05, Figures 5B,E–G). At the genus level, compared with the TY-S01 group, the abundance of *Escherichia–Shigella*, *Parasutterella*, *Clostridium-sensu-stricto-1*, and *Clostridioides* in the CM group was significantly increased while the abundance of unclassified-f-*Lachnospiraceae*, *Desulfovibrio*, *Eubacterium-brachy-*group and *Lachnospiraceae-*UCG-006 was significantly reduced (Figures 5H,I).

### Effect of TY-S01 on the Short-Chain Fatty Acids Content in Feces of Mice

**FIGURE 6:**
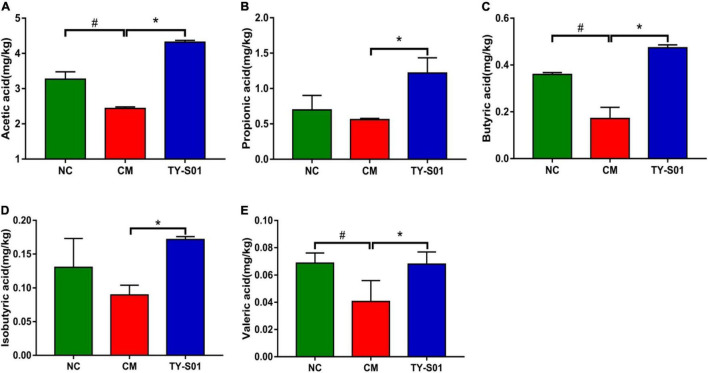
Effect of TY-S01 on the SCFAs content in feces of mice. The fecal levels of acetic acid **(A)**, propionic acid **(B)**, butyrate acid **(C)**, isobutyrate acid **(D)**, and valeric acid **(E)**. NC is the normal control group; CM is the constipation model group; TY-S01 is the treatment group with TY-S01. ^#^ represents the significant difference between the NC and CM groups (*p* < 0.05); * represents the significant difference between the CM and TY-S01 groups (*p* < 0.05). Data are presented as mean ± SD.

GC was utilized to determine the effect of TY-S01 on the content of SCFAs in feces. The acetic acid (AA), butyric acid (BA), and valeric acid (VA) were obviously decreased in the CM group compared to the NC group (*p* < 0.05, Figures 6A,C,E). Although the levels of propionic acid (PA) and isobutyric acid (IBA) also declined, there was no statistical difference (*p* < 0.05, Figures 6B,D). The levels of AA, PA, BA, IBA, and VA in the TY-S01 group were significantly higher compared to the CM group (*p* < 0.05, Figures 6A–E). Apparently, TY-S01 maintained the levels of SCFAs, which provided assistance to prevent constipation possibly.

### Correlations Analysis Between Gut Microbiota and Metabolic Biomarkers

**FIGURE 7:**
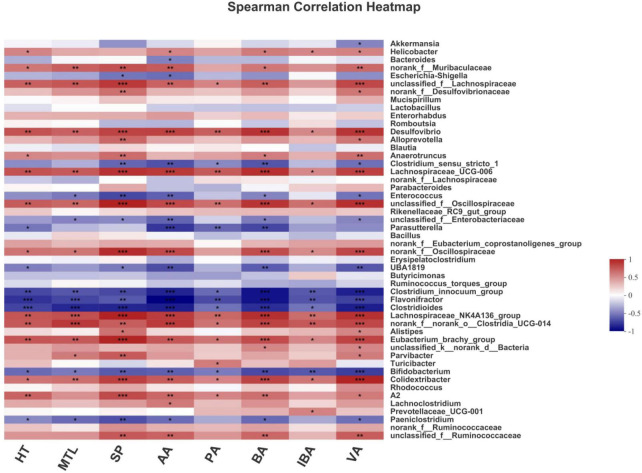
Correlation analysis between gut microbiota and metabolic biomarkers at the genus level. The color ranges from blue to red, indicating the change in correlation from negative to positive. HT is 5-hydroxytryptamine; MTL is motilin; SP is substance P; AA is acetic acid; PA is propionic acid; BA is butyrate acid; IBA is isobutyrate acid; VA is valeric acid. Asterisks *, **, and *** represent 0.01 < *p* ≤ 0.05, 0.001 < *p* ≤ 0.01, and *p* ≤ 0.001, respectively.

Spearman’s correlation analysis was used to clarify the association of metabolic biomarkers of constipation with the top 50 species at the genus level (Figure 7). *Lachnospiraceae-NK4A136-*group, *Desulfovibrio*, *Eubacterium-brachy-*group, and *lachnospiraceae-*UCG-006 were positively associated with metabolic biomarkers (5-HT, MTL, SP, AA, PA, BA, IBA, and VA). The unclassified-f-*Lachnospiraceae* was positively correlated with all of the above metabolic biomarkers but not IBA. The SP and AA were negative correlations with *Escherichia-Shigella* and *Clostridium-sensu-stricto-1* was negatively related to SP, AA, PA, BA, and VA. Moreover, the 5-HT, AA, PA, and BA were a negative association with *Parasutterella*. The above data indicated that the evolvement of constipation was closely associated with the gut microbiota.

## Discussion

Recently, probiotics have acquired plenty of attention owing to their efficacy and no side effects (17). In this investigation, we approved that *B. lactis* TY-S01 modulated gut microbiota and its metabolites to prevent loperamide-induced constipation in mice.

The FWC, time of FBS, and GTR are prerequisite indicators for judging the severity of constipation (18). The supply of loperamide incited reduced FWC, prolonged time to FBS, and decreased GTR in the current study. Our results confirmed the ability of TY-S01 to maintain stool humidity and accelerate intestinal peristalsis.

The damaged intestinal morphology could weaken the frequency and degree of the intestinal peristalsis (19). According to H&E staining, mice in the CM group displayed impaired epithelial integrity of the intestine, the disruption of intestinal villi, and the infiltration of inflammatory cells. Nevertheless, TY-S01 maintained the intestinal morphology to the criterion of the NC group. MUC-2 is mucin that covers intestinal epithelial cells to form a protective layer, which helps maintain the integrity of the intestinal mucosal barrier (20). The MUC-2 expression was increased obviously after the supplementation of TY-S01. Constipation is also correlated to impairment of the intestinal mucosal immune system, and animal models verified a causal relationship between the presence of mucosal inflammation and altered sensorimotor function (21). TY-S01 inhibited mRNA expression of pro-inflammatory mediators, including TNF-α, IL-6, and IL-1β. The reduced inflammatory response in the intestine would help avoid the destruction of tight junction proteins and the subsequent increase in intestinal permeability (22). The results indicated that TY-S01 maintained the integrity of gut barrier and attenuated intestinal inflammation.

5-Hydroxytryptamine is mainly distributed in the digestive tract and is secreted by enterochromaffin cells on intestinal mucosa (23). The release of 5-HT from enterochromaffin cells is one of the main triggers of motor and sensory reflex activity in the gut, so the concentration of 5-HT in serum can be used to determine the changes in intestinal function. As 5-HT enhances diarrhea while lessens in constipation symptoms. The elevation of 5-HT induce the release of gastrointestinal hormones such as SP and MTL (24). The SP and MTL are excitatory neuropeptides and associated with the acceleration of intestinal motility (25, 26). In our study, TY-S01 maintained the 5-HT, SP, and MTL at normal levels in constipated mice. Studies have confirmed that 5-HT needs to bind to specific receptors to function *in vivo*. 5-HT_4_R is one of the specific receptors for 5-HT, which is associated with enhanced intestinal propulsion and shortened intestinal transit time (27). Down-regulation of 5-HT_4_R expression was proposed in constipation patients (28). SERT is a transmembrane transporter and expresses on enterochromaffin membranes and presynaptic membranes devote to the transport of 5-HT secreted in the intestine (2). *Lactobacillus* could relieve constipation by up-regulating the SERT expression in intestinal epithelial cells (29). Aberrant expressions of 5-HT_4_R and SERT indicate gastrointestinal dysfunction (2). TY-S01 up-regulated the mRNA expressions of 5-HT_4_R and SERT remarkably, indicating that TY-S01 contributed to the normalization of intestinal function.

Short-chain fatty acids are the final metabolites of indigestible carbohydrates that are fermented by gut microbiota (including acetic, propionic, isobutyrate, butyrate, valeric, etc.), which promote the normalization of intestinal motility (30, 31). Several studies have indicated that probiotics ensure the levels of SCFAs in the gut, which maintains normal bowel movements (32). Different SCFAs have different divisions of labor in relieving constipation. As the foremost outcome of colonic fermentation, acetic accelerate the absorption of water and electrolytes to stimulate the peristalsis of the intestine (33). Butyrate may affect gut motility by regulating immune system-associated T-reg cells and limiting intestinal inflammatory response (34). Propionic, butyric, and valeric can ameliorate constipation by inducing colonic contractions and acting directly on smooth muscle (35). Our study confirmed that TY-S01 could make the level of SCFAs in constipated mice comparable to normal mice, which will be beneficial to prevent the occurrence of constipation.

Differences in gut microbiota between constipated patients and healthy individuals have been bought forward by numerous studies (36). Similarly, the NMDS displayed that the constipated mice had an unequal microbiota structure compared with normal mice in our study, while the microbiota structure of constipated mice was similar to normal mice after TY-S01 intervention. Chao and Shannon indexes are often used to represent the richness and diversity of the community. TY-S01 was able to maintain the richness and diversity of gut microbiota unaffected when loperamide was administered. We further examined the abundance of specific species in the gut with or without TY-S01 treatment. TY-S01 increased the ratio of *Firmicutes* to *Bacteroidetes*, which was consistent with the study by Wang et al. (15). Research indicated that the ratio of *Firmicutes* to *Bacteroidetes* was positively related to intestinal propulsion and negatively related to stool consistency (37). The abundance of *Proteobacteria* was detected to lower significantly in the TY-S01 group. *Proteobacteria* comprise many pathogenic genera and are believed to be a potential pro-inflammatory phylum (38). The positive association of some species with constipation was inhibited in constipated mice *via* the TY-S01 intervention, such as *Ruminococcaceae*, *Clostridium-sensu-stricto-1*, *Parasutterella*, and *Escherichia-Shigella* (12, 39, 40). Furthermore, the populations of *Lachnospiraceae*, *Bacteroidaceae*, *Desulfovibrio*, and *Eubacterium-brachy-*group were heightened after TY-S01 treatment. *Lachnospiraceae* facilitates the peristalsis of the intestine and the composite of 5-HT in enterochromaffin cells (41). *Bacteroidetes* promote the expression of gut-associated proteins to accelerate intestinal motility (42). And *Desulfovibrio, Eubacterium-brachy-*group have a positive effect on ameliorating constipation (12, 43). Surprisingly, there were obvious associations of metabolic biomarkers with specific species in the intestine by correlation analysis. Our findings suggested that TY-S01 could prevent gut dysbiosis caused by constipation and mediate the production of microbiota metabolites.

## Conclusion

Comprehensively, TY-S01 prevented loperamide-induced constipation in mice, involving the acceleration of intestinal peristalsis, the maintenance of feces humidity, the prevention of damaged gut barrier, and the prevention of intestinal dysfunction, and the repression of intestinal inflammatory response. The underlying mechanism was related to maintaining the balance of gut microbiota, accompanied by beneficial modulation of gut metabolites such as SCFAs, 5-HT, and gastrointestinal hormones. The results suggest that TY-S01 should be investigated for its ability to prevent or manage constipation in humans.

## Data Availability Statement

The data presented in this study were deposited in the NCBI repository, Submission ID is SUB11203385 and the BioProject ID is PRJNA817683. It can be accessed through the following link: http://www.ncbi.nlm.nih.gov/bioproject/817683.

## Ethics Statement

The animal study was reviewed and approved by the Experimental Animal Welfare Ethics Review Committee of Chongqing Institute of Traditional Chinese Medicine (5001087226041, Chongqing, China).

## Author Contributions

TT: methodology, investigation, and writing manuscript and editing. JW: methodology, visualization, and formal analysis. YJ and ZZ: resources. XZ, YW, XS, and YD: investigation. FZ: conceptualization, funding acquisition, and supervision. All authors read and approved the final manuscript.

## Conflict of Interest

TT, JW, YJ, XZ, ZZ, YW, XS, YD, and FZ were employed by the company Chongqing Tianyou Dairy Co., Ltd.

## Publisher’s Note

All claims expressed in this article are solely those of the authors and do not necessarily represent those of their affiliated organizations, or those of the publisher, the editors and the reviewers. Any product that may be evaluated in this article, or claim that may be made by its manufacturer, is not guaranteed or endorsed by the publisher.

## Footnotes

1
www.majorbio.com

## References

[1] Hanson B Siddique SM Scarlett Y Sultan S. American gastroenterological association institute technical review on the medical management of opioid-induced constipation. *Gastroenterology.* (2019) 156:229–53. 10.1053/j.gastro.2018.08.018 30337104 PMC6685294

[2] Lu YY Zhang JX Zhang Z Liang X Liu TJ Yi HX Konjac glucomannan with probiotics acts as a combination laxative to relieve constipation in mice by increasing short-chain fatty acid metabolism and 5-hydroxytryptamine hormone release. *Nutrition.* (2020) 84:111112. 10.1016/j.nut.2020.111112 33454530

[3] Guo M Yao J Yang F Liu W Bai H Ma J The composition of intestinal microbiota and its association with functional constipation of the elderly patients. *Future Microbiol.* (2020) 15:163–75. 10.2217/fmb-2019-0283 32079430

[4] Bassotti G Antonelli E Villanacci V Salemme M Coppola M Annese V. Gastrointestinal motility disorders in inflammatory bowel diseases. *World J Gastroenterol.* (2014) 20:37–44. 10.3748/wjg.v20.i1.37 24415856 PMC3886030

[5] Brierley SM Linden DR. Neuroplasticity and dysfunction after gastrointestinal inflammation. *Nat Rev Gastroenterol Hepatol.* (2014) 11:611–27. 10.1038/nrgastro.2014.103 25001973

[6] D’Antongiovanni V Pellegrini C Fornai M Colucci R Blandizzi C Antonioli L Intestinal epithelial barrier and neuromuscular compartment in health and disease. *World J Gastroenterol.* (2020) 26:1564–79. 10.3748/wjg.v26.i14.1564 32327906 PMC7167418

[7] Moreira TR Leonhardt D Conde SR. Influence of drinking a probiotic fermented milk beverage containing *Bifidobacterium animalis* on the symptoms of constipation. *Arq Gastroenterol.* (2017) 54:206–10. 28591244 10.1590/S0004-2803.201700000-27

[8] Costedio MM Coates MD Brooks EM Glass LM Ganguly EK Blaszyk H Mucosal serotonin signaling is altered in chronic constipation, but not in opiate-induced constipation. *Am J Gastroenterol.* (2010) 105:1173. 10.1038/ajg.2009.683 20010921 PMC2872481

[9] Bowersox S Clark R Ellis DJ Palme M Sims E Druzgala P. Molecular pharmacology of naronapride, a selective 5-HT4 receptor agonist for the treatment of constipation: comparison with other prokinetic 5-HT4 receptor agonists. *Gastroenterol.* (2011) 140:S612. 10.1016/S0016-5085(11)62531-6

[10] Dimidi E Christodoulides S Fragkos KC Scott SM Whelan K. The effect of probiotics on functional constipation in adults: a systematic review and meta-analysis of randomized controlled trials. *Am J Clin Nutr.* (2014) 100:1075–84. 10.3945/ajcn.114.089151 25099542

[11] Agrawal A Houghton LA Morris J Reilly B Guyonnet D Goupil FN Clinical trial: the effects of a fermented milk product containing *Bifidobacterium lactis* DN-173010 on abdominal distension and gastrointestinal transit in irritable bowel syndrome with constipation. *Aliment Pharmacol Ther.* (2009) 29:104–14. 10.1111/j.1365-2036.2008.03853.x 18801055

[12] Yang ZD Ye SM Xu ZM Su HH Tian X Han B Dietary synbiotic ameliorates constipation through the modulation of gut microbiota and its metabolic function. *Food Res Int.* (2021) 147:110569. 10.1016/j.foodres.2021.110569 34399543

[13] Wang L Hu L Xu Q Jiang T Fang S Wang G Bifidobacteria exert species-specific effects on constipation in BALB/c mice. *Food Funct.* (2017) 8:3587–600. 10.1039/c6fo01641c 28884754

[14] Wang R Sun J Li G Zhang M Niu T Kang X Effect of *Bifidobacterium animalis* subsp. *lactis* MN-Gup on constipation and the composition of gut microbiota. *Benef Microbes.* (2021) 12:31–42. 10.3920/BM2020.0023 33308038

[15] Wang L Hu L Yan S. Effects of different oligosaccharides at various dosages on the composition of gut microbiota and short-chain fatty acids in mice with constipation. *Food Funct.* (2017) 8:1966–78. 10.1039/c7fo00031f 28475191

[16] Moreau NM Goupry SM Antignac JP Monteau FJ Le Bizec BJ Champ MM Simultaneous measurement of plasma concentrations and 13C-enrichment of short-chain fatty acids, lactic acid and ketone bodies by gas chromatography coupled to mass spectrometry. *J Chromatogr B.* (2003) 784:395–403. 10.1016/S1570-0232(02)00827-9 12505787

[17] Dimidi E Christodoulides S Scott SM Whelan K. Mechanisms of action of probiotics and the gastrointestinal microbiota on gut motility and constipation. *Adv Nutr.* (2017) 8:484–94. 10.3945/an.116.014407 28507013 PMC5421123

[18] Li G Wang Q Qian Y Zhou Y Wang R Zhao X. Component analysis of Pu-erh and its anti-constipation effects. *Mol Med Rep.* (2014) 9:2003–9. 10.3892/mmr.2014.2009 24604453

[19] Setchell KD Brown NM Zimmer-Nechemias L Brashear WT Wolfe BE Kirschner AS Evidence for lack of absorption of soy isoflavone glycosides in humans, supporting the crucial role of intestinal metabolism for bioavailability. *Am J Clin Nutr.* (2002) 76:447–53. 10.1051/rnd:2002034 12145021

[20] Wlodarska M Thaiss CA Nowarski R. NLRP6 inflammasome orchestrates the colonic host-microbial interface by regulating goblet cell mucus secretion. *Cell.* (2014) 156:1045–59. 10.1016/j.cell.2014.01.026 24581500 PMC4017640

[21] Collins SM. The immunomodulation of enteric neuromuscular function: implications for motility and inflammatory disorders. *Gastroenterology.* (1996) 111:1683–99. 10.1016/S0016-5085(96)70034-3 8942751

[22] Zhou Q Costinean S Croce CM Brasier AR Merwat S Larson SA MicroRNA 29 targets nuclear factor-kappaB-repressing factor and claudin1 to increase intestinal permeability. *Gastroenterology.* (2015) 148:158–69. 10.1053/j.gastro.2014.09.037 25277410 PMC4303568

[23] Racke K Reimann A Schworer H Kilbinger H. Regulation of 5-HT release from enterochromaffin cells. *Behav Brain Res.* (1996) 73:83–7. 8788482 10.1016/0166-4328(96)00075-7

[24] Zhu XW Liu ZB Qu HY Niu WM Gao L. The effect and mechanism of electroacupuncture at LI11 and ST37 on constipation in a rat model. *Acupunct Med.* (2016) 34:194–200. 10.1136/acupmed-2015-010897 26561562 PMC4941155

[25] Suo HY Zhao X Qian Y Li GJ Liu ZH Xie J Therapeutic effect of activated carbon-induced constipation mice with *Lactobacillus fermentum* Suo on treatment. *Int J Mol Sci.* (2014) 15:21875–95. 10.3390/ijms151221875 25464378 PMC4284683

[26] Tzavella K Riepl RL Klauser AG. *Eur J Gastroenterol Hepatol.* (1996) 8:1207–11. 10.4103/2230-973X.82383 8980942

[27] Lacy BE Yu SY. Tegaserod: a new 5-HT4 agonist. *J Clin Gastroenterol.* (2002) 34:27–33. 10.1097/00004836-200201000-00006 11743242

[28] Malagelada C Nieto A Mendez S Accarino A Santos J Malagelada JR Effect of prucalopride on intestinal gas tolerance in patients with functional bowel disorders and constipation. *J Gastroenterol Hepatol.* (2017) 32:1457–62. 10.1111/jgh.13733 28090679

[29] Cao YN Feng LJ Wang BM Jiang K Li S Xu X *Lactobacillus acidophilus* and *Bifidobacterium longum* supernatants upregulate the serotonin transporter expression in intestinal epithelial cells. *Saudi J Gastroenterol.* (2018) 24:59–66. 10.4103/sjg.SJG_333_17 29451186 PMC5848327

[30] Morrison DJ Preston T. Formation of short chain fatty acids by the gut microbiota and their impact on human metabolism. *Gut Microbes.* (2016) 7:189–200. 10.1080/19490976.2015.1134082 26963409 PMC4939913

[31] Cherbut C Ferrier L Rozé C Anini Y Galmiche JP. Short-chain fatty acids modify colonic motility through nerves and polypeptide YY release in the rat. *Am J Physiol.* (1998) 275:1415–22. 10.1063/1.4897078 9843779

[32] Salminen S Salminen E. Lactulose, lactic acid bacteria, intestinal microecology and mucosal protection. *Scand J Gastroenterol.* (1997) 32:45–8. 10.1080/00365521.1997.11720717 9145446

[33] Smith PM Howitt MR Panikov N Michaud M Gallini CA Bohlooy-Y M The microbial metabolites, short-chain fatty acids, regulate colonic T-reg cell homeostasis. *Science.* (2013) 341:569–73. 10.1126/science.1241165 23828891 PMC3807819

[34] Kieffer DA Martin RJ Adams SH. Impact of dietary fibers on nutrient management and detoxification organs: gut, liver, and kidneys. *Adv Nutr.* (2016) 7:1111–21. 10.3945/an.116.013219 28140328 PMC5105045

[35] Rondeau MP Meltzer K Michel KE. Short chain fatty acids stimulate feline colonic smooth muscle contraction. *J Feline Med Surg.* (2003) 5:167–73. 10.1016/s1098-612x(03)00002-0 12765627 PMC10822495

[36] Mancabelli L Milani C Lugli GA. Unveiling the gut microbiota composition and functionality associated with constipation through metagenomic analyses. *Sci Rep.* (2017) 7:9879. 10.1038/s41598-017-10663-w 28852182 PMC5575163

[37] Hollister EB Cain KC Shulman RJ Jarrett ME Burr RL Ko C Relationships of microbiome markers with extraintestinal, psychological distress and gastrointestinal symptoms, and quality of life in women with irritable bowel syndrome. *J Clin Gastroenterol.* (2020) 54:175–83. 10.1097/MCG.0000000000001107 30148765 PMC6387862

[38] Sheng Y Liu J Zheng S Liang F Luo Y Huang K Mulberry leaves ameliorate obesity through enhancing brown adipose tissue activity and modulating gut microbiota. *Food Funct.* (2019) 10:4771–81. 10.1039/C9FO00883G 31312821

[39] Engevik MA Luk B Chang-Graham AL Hall A Herrmann B Ruan W Bifidobacterium dentium fortifies the intestinal mucus layer *via* autophagy and calcium signaling pathways. *mBio.* (2019) 10:3. 10.1128/mBio.01087-19 31213556 PMC6581858

[40] Guerra-Ordaz AA González-Ortiz G La Ragione RM Woodward MJ Collins JW Pérez JF Lactulose and *Lactobacillus plantarum*, a potential complementary synbiotic to control postweaning colibacillosis in piglets. *Appl Environ Microbiol.* (2014) 80:4879–86. 10.1128/AEM.00770-14 24907322 PMC4135760

[41] Taur Y Pamer EG. Harnessing microbiota to kill a pathogen: fixing the microbiota to treat clostridium difficile infections. *Nat Med.* (2014) 20:246–7. 10.1038/nm.3492 24603796 PMC4542075

[42] Hooper LV Wong MH Thelin A Hansson L Falk PG Gordon JI. Molecular analysis of commensal host-microbial relationships in the intestine. *Science.* (2001) 291:881–4. 10.2307/3082402 11157169

[43] Zhang XY Zheng JP Jiang N Sun GG Bao XK Kong MW Modulation of gut microbiota and intestinal metabolites by lactulose improves loperamide-induced constipation in mice. *Eur J Pharm Sci.* (2021) 158:105676. 10.1016/j.ejps.2020.105676 33310029

